# Novel human reovirus isolated from children and its long-term circulation with reassortments

**DOI:** 10.1038/s41598-020-58003-9

**Published:** 2020-01-22

**Authors:** Seiji P. Yamamoto, Daisuke Motooka, Kazutaka Egawa, Atsushi Kaida, Yuki Hirai, Hideyuki Kubo, Kazushi Motomura, Shota Nakamura, Nobuhiro Iritani

**Affiliations:** 1 Division of Microbiology, Osaka Institute of Public Health, Osaka, 543-0026 Japan; 2 Research Institute for Microbial Diseases, Osaka University, Osaka, 565-0871 Japan; 3 Division of Public Health, Osaka Institute of Public Health, Osaka, 537-0025 Japan

**Keywords:** Viral epidemiology, Viral pathogenesis

## Abstract

Mammalian orthoreovirus (MRV), also known as reovirus, was discovered in the 1950s and became the first reported segmented double-stranded RNA virus. MRVs have since been found in a variety of animal species, including humans. However, reports on MRV infections are scarce due to the rarity of their symptomatic occurrence. In Japanese surveillance studies, MRVs have been detected as gastrointestinal pathogens since 1981, with a total of 135 records. In Osaka City, Japan, MRV was first isolated in 1994 from a child with meningitis, and then in 2005 and 2014 from children with gastroenteritis. Here, we conducted the first molecular characterization of human MRV isolates from Japan and identified a novel human reovirus strain belonging to MRV type 2, designated the MRV-2 Osaka strain. This strain, with all three isolates classified, is closely related to MRV-2 isolates from sewage in Taiwan and is relatively close to an MRV-2 isolate from a bat in China. Our data suggest that the MRV-2 Osaka strain, which has circulated amongst humans in Japan for at least two decades, has spread internationally.

## Introduction

The mammalian orthoreovirus (MRV) species belongs to the genus *Orthoreovirus* of the family *Reoviridae*, and is characterized by 10 segments of double-stranded RNA (dsRNA) genome encapsulated in the viral particles with a diameter of 65 to 80 nm^1^. The ten dsRNA segments consist of three large (L1–L3), three medium (M1–M3), and four small (S1–S4) size class segments. The S1 segment encodes an attachment protein that binds to host cell receptors, thereby determining cell and tissue tropism, as well as the virus serotype^1^. Four virus strains isolated from children in the 1950s (type 1 Lang, type 2 Jones, type 3 Abney, and type 3 Dearing) are the most frequently used MRV-1–3 prototypes to date. The fourth serotype (MRV-4) was isolated from a mouse, but its infection in humans has never been reported^2^.

Due to the segmented nature of MRV genomes, they are capable of undergoing gene reassortment during co-infection in one cell, resulting in the emergence of reassortants containing mixed segments from two or more different parental strains. Since there has been no type-specific segment found, except for the S1 segment, it seems that segments can be exchanged between any types of MRV.

MRV has a wide geographic distribution, and is considered to infect practically all mammals, including humans^1^. Human infections with MRV are common in early childhood^3,4^, but are rarely symptomatic^1^. When MRV produces symptoms in human, the most common manifestations are coryza, pharyngitis, cough^5^, as well as gastroenteritis^6^. More severe cases, including neurological diseases and meningitis, have also been reported^6–10^. However, MRV is rarely detected in humans, even during longitudinal studies^6,11^, although it is one of the most abundant viruses in environmental waters^11,12^.

Here, we present epidemiological information regarding MRV detections in Japan since 1981. We conducted the first molecular characterization of MRV isolates from children in Japan, and found that all isolates are a novel human reovirus strain, designated MRV-2 Osaka. We also provide evidence that the MRV-2 Osaka strain has undergone multiple reassortment events during its decades of circulation.

## Results

### Circulation of MRV in Japan

**Figure 1:**
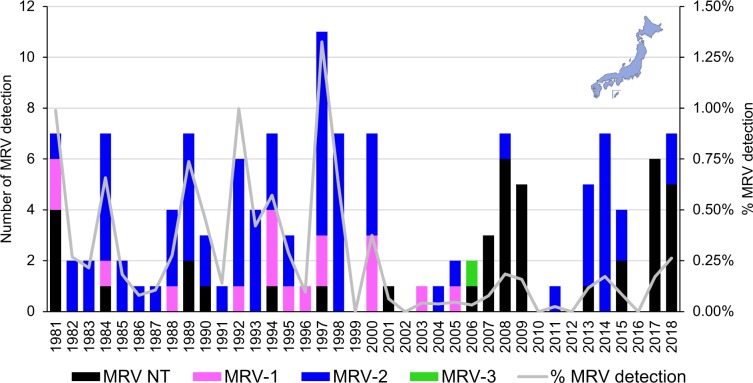
Yearly distribution of mammalian orthoreovirus (MRV) detected as a gastrointestinal pathogen between 1981 and 2018 in Japan. The data were retrieved from Infectious Agents Surveillance Report, which is publicly available on the website of the National Institute of Infectious Diseases of Japan. NT denotes not typed.

We retrieved data of MRV detection in Japan from the Infectious Agents Surveillance Report (IASR), which shows the yearly distribution of MRV detected as a gastrointestinal pathogen (Fig. 1). Between 1981 and 2018, a total of 135 cases of MRV were detected from human sources in Japan and were reported to IASR. MRV accounted for up to 1.3% (11 detections in 1997) and an average of 0.3% (3.6 detections per year) of gastrointestinal viruses. MRV detection was not recorded in 1999, 2002, 2010, 2012, and 2016. MRV-2 was detected throughout the period, and was the most prevalent type (77, 57.0%), followed by MRV-1 (17, 12.6%) and MRV-3 (1, 0.7%), and 40 isolates (29.6%) were not typed. There were data limitations. It is not clear how these MRVs were detected and typed. It is not clear whether each MRV was single-detected or co-detected with other gastroenteritis viruses. Finally, no patient data is available, such as symptoms other than gastroenteritis, age and sex distributions, and underlying medical conditions. However, it must be noted that MRVs have been continuously detected from humans in Japan since 1981, suggesting that there might be certain MRV strains endemic to Japan.

### Isolation of MRV

In the virus surveillance system in Osaka City, Japan, we isolated three MRV strains. The first strain, Osaka1994, was isolated in November 1994 using MA-104 cells from a stool specimen of a 4-month-old boy who had aseptic meningitis and fever (40 °C). The second strain, Osaka2005, was isolated in July 2005 using Vero cells from a stool specimen of a 10-month-old boy who had diarrhea along with bloody stool and fever (38 °C). The third strain, Osaka2014, was isolated in May 2014 using Vero cells from a stool specimen of a 6-year-old boy with an underlying acute lymphocytic leukemia who had diarrhea and abdominal pain. The third specimen tested negative for norovirus GI and GII, rotavirus A, adenovirus 40 and 41, human sapovirus, human astrovirus, and human parechovirus. The reovirus-like particles in each sample of isolation medium were identified by transmission electron microscopy, and subjected to further identification tests.

### Molecular characterization of MRV isolates

**Figure 2:**
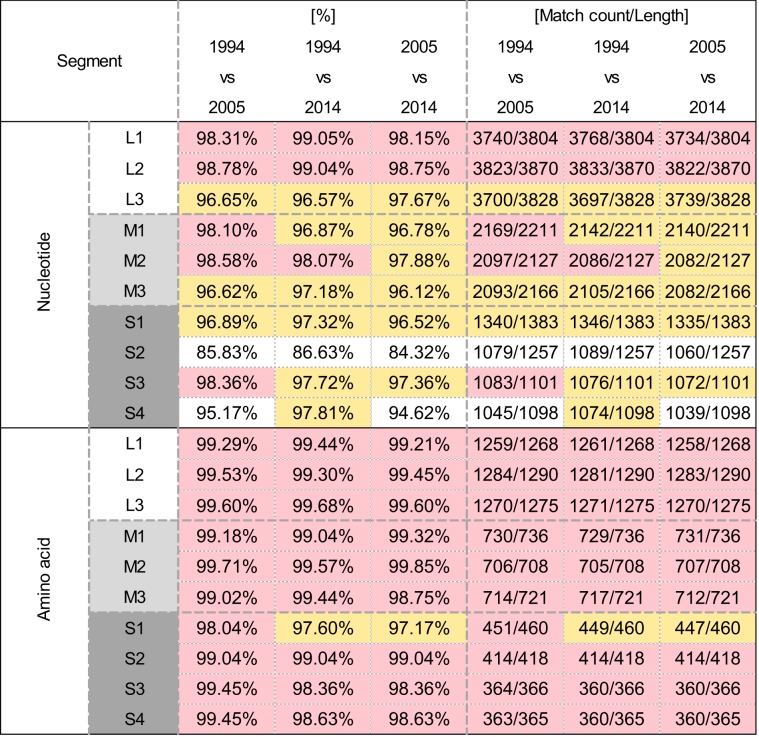
Nucleotide and amino acid sequence identities between novel human reovirus strains detected in 1994, 2005, and 2014 in Osaka City, Japan. Each sequence length is the full-open reading frame region determined by the Illumina MiSeq platform. Pink indicates ≥98% identity between strains. Yellow indicates ≥96% identity between strains.

**Figure 3:**
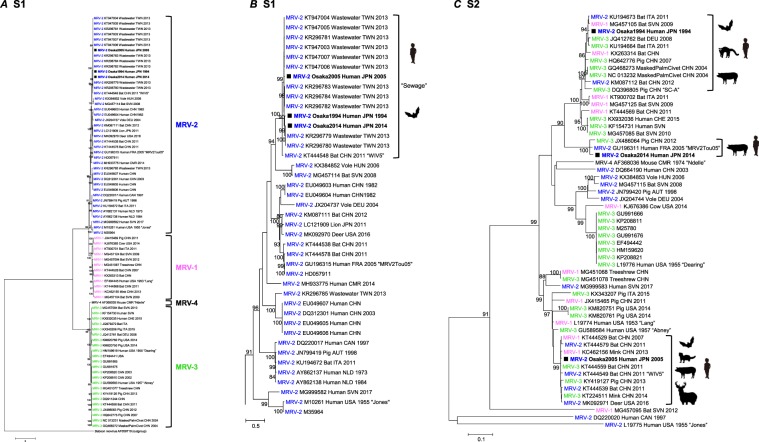
Phylogenetic analysis based on the nucleotide sequence of the MRV-2 Osaka strains. (**A**) Maximum-likelihood phylogram based on partial nucleotide sequences of the S1 segment (MRV-2 Osaka strains, 1,023 bp). The MRV-2 branch is enlarged in (**B**). (**C**) Maximum-likelihood phylogram based on partial nucleotide sequences of the S2 segment (MRV-2 Osaka strains, 1,155 bp). Solid squares indicate the strains isolated in this study. Each bracket indicates a cluster that includes the strain isolated in this study. Each strain ID consisting of MRV type, accession number, host species, three-letter country name abbreviation, and detection year is based on data obtained from GenBank in February 2019. The names of MRV prototype strains and other important strains to this study are enclosed in double quotes and presented at the end of each strain ID. On the basis of Bayesian information criteria, a general time reversible plus gamma plus invariable sites model was used to construct the phylogram. Numbers at the nodes indicate the bootstrap support values, which are given as a percentage of 1,000 replicates (values less than 80% are omitted). Scale bar indicates genetic distances (nucleotide substitutions per site). Country abbreviations include: AUT, Austria; CAN, Canada; CHE, Switzerland; CHN, China; CMR, Cameroon; DEU, Germany; FRA, France; HUN, Hungary; ITA, Italy; JPN, Japan; KOR, South Korea; NLD, The Netherlands; RUS, Russia; SVN, Slovenia; TWN, Taiwan; and USA, United States of America.

Our initial attempts to amplify the S1 segment of the MRV isolates using originally designed MRV specific primers failed. Thus, we employed the Illumina MiSeq platform to read sequences without any sequence-dependent primers. This approach successfully determined nucleotide sequences of all 10 segments of each isolate. Phylogenetic analysis of the S1 segment showed that the three MRV isolates were classified as MRV-2 and indicated that isolates possess 96.5–97.3% nucleotide and 97.1–98.0% amino acid identities with each other (Fig. 2), and belonged to the same cluster as the 11 strains detected from the sewage in Taiwan^12^ and a WIV5 strain detected from a *Hipposideros* bat in China^13^ (Fig. 3A,B). In the region of the S1 segment used for the phylogenetic analysis, the three MRV-2 isolates showed 95.9–99.8% and 91.3–93.4% nucleotide identity to the MRV-2 strains from Taiwan and the WIV5 strain from China, respectively. The sequences of the S1 segment of this cluster showed great diversity at the nucleotide and amino acid levels (less than 80% identity), compared with the S1 segment from other MRV-2 strains. This indicates that the three isolates can be considered as a novel human reovirus strain, referred to as the MRV-2 Osaka strain, based on the presence of an Osaka1994-like S1 protein. The MRV-2 Osaka strain has been circulating in Japan as well as in East Asian countries for at least two decades.

Comparisons of each segment among the three MRV-2 isolates demonstrated that the S2 segments, encoding a major inner-capsid protein, showed particularly low nucleotide identity (less than 87%) but high amino acid identity (99.0%) to each other (Fig. 2). Phylogenetic analysis revealed that the S2 segment of each MRV-2 Osaka strain belonged to a different cluster (Fig. 3C). The Osaka1994 strain clustered with 96.2–99.0% nucleotide identity with MRV-1–3 strains (including an MRV-3 SC-A strain) from bats (China, Germany, Italy, and Slovenia), pigs (China), and masked palm civets (China). Similarly, the S2 segment of the Osaka2005 strain was closely related to that of MRV-1–3 strains isolated from the bats (China), minks (China), a pig (China), and a white-tailed deer (United States of America) with 98.5–99.6% nucleotide identity, and shared a relatively high identity (95.5%) with that of the prototype MRV-3 Abney strain (Fig. 3C). MRV strains with S2 segment closely related to that of the Osaka1994 or Osaka2005 strains were isolated from animals other than humans, whereas the S2 segment of the Osaka2014 showed the highest nucleotide identity (97.7%) to an MRV2Tou05 strain detected in children with encephalopathy in France^10^, followed by a swine MRV-3 strain (96.9%) detected in China (Fig. 3C). The other eight segments of the three MRV-2 Osaka strains were closely related to each other at the nucleotide and amino acid levels except for the S4 segment of the Osaka2005 strain sharing a slightly low nucleotide identity with the Osaka1994 and Osaka2014 strains (Fig. 2 and Supplementary Fig. S1). Taken together, our data suggest that the MRV-2 Osaka strain has undergone multiple reassortment events during the decades of its circulation.

## Discussion

Since its discovery during the 1950s in the USA, MRVs have been isolated from humans and from other animals and environmental waters. Seroepidemiological surveys revealed that reovirus infections occur early in childhood in most humans^3,4^. We showed the yearly distribution of MRV reported in IASR as a gastrointestinal pathogen in Japan, between 1981 and 2018. MRV-2, MRV-1, and MRV-3 accounted for 57%, 13%, and 0.7% of the cases, respectively. In Japan, MRV isolation from humans was first reported in 1962 by Moritsugu *et al*.^13^. Moreover, MRV-2 was the most detected type from 1962 to 1967, followed by MRV-1, while MRV-3 was not found during the study period^14^. Thus, MRV-2 has long been the most prevalent type among humans in Japan, followed by MRV-1, whereas MRV-3 is rare^9,14^. Nevertheless, molecular characterization of the MRVs detected from the Japanese patients has not yet been performed. Here, we sequenced ten segments of three MRV isolates, each of which was isolated in 1994, 2005, and 2014. Surprisingly, even though they were at most 20 years apart, all three strains possessed a very similar S1 segment, and were considered as a novel human reovirus, designated the MRV-2 Osaka strain, because the S1 segment of our isolates was phylogenetically separate from that of any known human reovirus. Similar MRV-2 strains with S1 segment sequences available in the NCBI Nucleotide database were isolated across the island of Taiwan from wastewater samples in an environmental surveillance in Taiwan during 2012 and 2013^12^. Interestingly, the MRV type distribution in Taiwanese sewage was predominantly MRV-2 while MRV-3 was rare, similar the MRV type distribution in humans in Japan. Since MRV type distributions among humans can be reflected in the detection pattern from environmental water due to human excretion^9^, MRV-2 may have infected people across Taiwan in the same manner as the MRV-2 Osaka strain, although no cases of MRV infection have been reported in Taiwan^12^. The other related strain, WIV5, was isolated from a bat in 2011 in China^13^. Bats are natural reservoirs of a variety of viruses, including reoviruses, and accumulating evidence indicates that the interspecies transmission of other orthoreovirus species from bats to humans seems to be frequent^15–18^. Therefore, the ancestral strain of the MRV-2 Osaka strain may have originated in bats, and subsequently retained in human population for a long time.

MRV can infect virtually all mammals, including humans. In spite of its ubiquity, the detailed molecular characterization of circulating MRVs has not been performed. The number of sequences in the NCBI Nucleotide database covering complete or near-complete ORF of each segment is less than 100, except for the S1 segment. One reason is probably because determining the sequence of a segmented genome is laborious. Another possible reason is the difficulty in amplifying the diversified S1 segment of MRV; our attempt to do so initially by conventional RT-PCR failed. Phylogenetic analysis revealed that each S2 segment of the three MRV-2 Osaka strains was unique, and formed a cluster with strains isolated from different host species and countries, and during different years, indicating that the MRV-2 Osaka strain has exchanged gene segments probably by circulating globally or by reassortments with some MRV strains imported to Japan. Therefore, considering its longitudinal and worldwide circulation among humans, the MRV-2 Osaka strain would have been characterized earlier, in the absence of difficulties in determining the S1 segment sequence. We eventually utilized the Illumina MiSeq platform to obtain sequences of the S1 segment as well as that of the other nine segments. The WIV5 strain, which belonged to the same cluster as the MRV-2 Osaka strain in the S1 segment, was also sequenced in a specific primer-independent manner using the 454/Roche sequencing platform^13^. It is noteworthy that Lim *et al*. successfully determined the related sequences using conventional RT-PCR and sequencing^12^. Their methods, which utilized sequence-specific primer pairs, could be useful for characterizing the S1 segment of strains related to the MRV-2 Osaka strain, improving understanding of its global distribution.

MRV infections are thought to be common before adulthood^3,4^. However, the reason for their infrequent association with disease remains an enigma. We isolated the Osaka MRV-2 strain from a child with meningitis and two children with gastroenteritis. Compared with those of MRV-1 and MRV-3, the neurotropism and neuropathology of MRV-2 are not well characterized. However, MRV-2 can cause nonlethal encephalitis in suckling mice^19^ and is capable of infecting neurons^20^. In humans, MRV-2 has been detected from cerebrospinal fluid of patients with neurological symptoms^9,10,21^. Therefore, MRV-2 could possibly be a causative agent in the meningitis case in this study. In the gastroenteritis case, since the child in 2014 had an underlying acute lymphocytic leukemia, he was probably in the state of reduced immunity, and thus was more vulnerable to virus infection. We confirmed that his stool specimen was negative for major gastroenteritis viruses, including norovirus and rotavirus, suggesting that his gastrointestinal symptoms were due to MRV-2 infection.

The reovirus traits of segmented genome, a very broad host range, and capability of infecting both respiratory and intestinal tracts, remind us of influenza A viruses (IAVs), which replicate in the intestine of the wild waterfowl and in the upper respiratory tracts of mammals, including humans. While IAVs are pathogenic to many mammalian species but have determinants of host rage restriction in their genome (e.g. haemagglutinin gene)^22^, the pathogenicity of MRVs can be mild in other species besides humans, and there is no evidence that any of the gene segments determine the host range of MRVs^1^. The low pathogenicity of MRVs is in stark contrast to the high pathogenicity of other members of the *Reoviridae*: rotaviruses annually cause the diarrhea-related deaths of 200,000 children in developing countries^23^ and orbiviruses, which include African horse sickness virus and Bluetongue virus, are infamous for having historically killed thousands to millions of animals per outbreak^24^. These facts indicate that MRVs may have achieved efficient viral replication in any mammalian species without debilitating them, which is likely to be an ideal strategy for a zoonotic virus to perpetuate virus species globally. But whether MRVs will become highly pathogenic in the future is unknown. Since MRVs can change drastically via gene reassortment events, monitoring and characterizing the circulating or emerging MRV strains should be done. Our study serves as a basis for epidemiology studies of MRVs that establish infections that we are as yet unaware of.

In summary, we demonstrate that symptomatic MRV infections in humans have been continuously and systematically reported in Japan since 1981, and identified a novel human reovirus designated the MRV-2 Osaka strain, which has circulated in Japan for at least two decades. Considering the reports of the detection of related strains in other countries besides Japan, the MRV-2 Osaka strain has spread internationally and may be prevalent in humans. This illustrates that we might still be unaware of the features of viruses that are human pathogens.

## Material and Methods

### Data of MRV detection in Japan

The data of MRV detection in Japan were retrieved from the IASR, which is publicly available at the website of the National Institute of Infectious Diseases of Japan (http://www.niid.go.jp/).

### Clinical sample collection

As part of our contribution to the National Epidemiological Surveillance of Infectious Diseases (NESID), Japan, outlined in the Infectious Diseases Control Law, clinical stool specimens were obtained from patients in a virus surveillance system in Osaka City^25^. Ten percent stool suspensions were subjected to virus isolation tests. MRV-2 Osaka1994 strain was isolated after three passages in MA-104 cells and was stored at −80 °C as the original stock in 1994. The MRV-2 Osaka2005 and Osaka2014 strains were isolated after two and five passages, respectively, in Vero cells and were stored at −80 °C as the original stock in 2005 and 2014, respectively.

### Negative-contrast electron microscopy

Transmission electron microscopy analysis was carried out as described previously^26^.

### Testing for gastroenteritis viruses

To test rotavirus A and adenovirus 40 and 41, 10% stool suspensions were subjected to ELISA by Rotaclone and Adenoclone E (TFB, Tokyo, Japan), respectively. Norovirus was tested by real-time RT-PCR as described previously^27^. Sapovirus, human astrovirus, and human parechovirus were tested by multiplex real-time RT-PCR using a QuantiTect multiplex PCR kit (QIAGEN, Hilden, Germany) and primer/probe sets as described previously^28^ with a modification of the sapovirus primers/probes^29^.

### Virus propagation and RNA sample preparation

Vero cells seeded on a 48-well plate were infected with each MRV isolate in the original stocks, and cultures were scaled up to five dishes with a diameter of 10 cm through three passages. This may have created cell culture-adaptive mutations in the MRV genes. Supernatant of infected Vero cells was clarified by centrifugation at 300* g* for 5 min at room temperature. Clarified supernatant was then filtrated through a 0.45 µm pore size membrane to remove cell debris. Filtrated supernatant was then ultracentrifuged at 163,000 *g* at 4 °C for 1 h. Viral RNA was recovered from pellet by using a TRIzol LS reagent (Thermo Fisher Scientific, Waltham, MA, USA), and was subsequently treated by a single-stranded RNA-specific RNase If (New England Biolabs, Ipswich, MA, USA) and DNase I (TaKaRa Bio, Shiga, Japan) to remove cellular RNA and genomic DNA, respectively. Finally, viral RNA was purified using phenol/chloroform extraction.

### Sequencing using the Illumina MiSeq platform

Viral RNA for each sample was sheared to about 200–300 bp using the model S220 device (Covaris, Woburn, MA, USA). After the denaturation of dsRNA at 95 °C for 3 min, each library was prepared using SMARTer Stranded Total RNA-Seq Kit v2 - Pico Input Mammalian (TaKaRa Bio and Clontech, Mountain View, CA, USA) according to manufacturer’s protocol. Paired -end sequencing (150 bp) was performed using a MiSeq v2 300 cycle kit (Illumina, San Diego, CA, USA). Raw reads were assembled using CLC Genomics Workbench (QIAGEN).

### Phylogenetic and homology analyses

MRV nucleotide sequences were obtained from the NCBI Nucleotide database. Although a total of 921 MRV nucleotide sequences were available as of February 9, 2019, sequences which did not cover at least the near-complete ORF were excluded from phylogenetic analyses. In the S1 analysis, all the S1 sequences of the MRV-2 strains and of the MRV-1, MRV-3, and MRV-4 strains, for which S2 sequences were available in the S2 analysis, were extracted from the dataset and used. In the analyses of the other segments, sequences of each segment were extracted from the dataset and used. Nucleotide alignment was confirmed by MUSCLE^30^ in MEGA 7.0 software^31^. On the basis of Bayesian information criteria, the optimal evolutionary model that best fit each sequence data set was identified, and the construction of a Maximum-likelihood tree with 1,000 bootstrap replications was performed using MEGA 7.0 software^31^. GENETYX Ver. 13 (GENETYX, Tokyo, Japan) was used to calculate nucleotide and amino acid homology.

### Ethical approval

All methods were carried out in accordance with relevant guidelines and regulations. Since clinical sample collection and the detection and characterization of pathogens were conducted under the Infectious Diseases Control Law in Japan, the ethics committee of the Osaka Institute of Public Health, which approved this study (No. 1709-08-3), waived the need to obtain informed consent for this study.

## Supplementary information

Supplementary information

## Data Availability

The GenBank/EMBL/DDBJ accession numbers for the sequences of the MRV-2 Osaka1994, Osaka2005, and Osaka2014 strains determined in this study are LC476895–LC476904, LC476905–LC476914, and LC476915–LC476924, respectively. Other datasets generated or analyzed during the current study are available from the corresponding author upon reasonable request.
