# Supplementary material for: Novel human reovirus isolated from children and its long-term circulation with reassortments
Source: Sci Rep. 2020 Jan 22;10:963. doi: 10.1038/s41598-020-58003-9 (PMC6976588; doi:10.1038/s41598-020-58003-9)

Title

Novel human reovirus isolated from children and its long-term circulation with reassortments

Authors

Seiji P. Yamamoto[a,*], Daisuke Motooka[b], Kazutaka Egawa[a], Atsushi Kaida[a], Yuki Hirai[a], Hideyuki Kubo[a], Kazushi Motomura[a], Shota Nakamura[b], and Nobuhiro Iritani[c]

Author Affiliations

[a] Division of Microbiology, Osaka Institute of Public Health, Osaka 543-0026, Japan
[b] Research Institute for Microbial Diseases, Osaka University, Osaka 565-0871, Japan
[c] Division of Public Health, Osaka Institute of Public Health, Osaka 537-0025, Japan

*Corresponding author: Seiji P. Yamamoto, Ph.D.
Osaka Institute of Public Health, 8-34 Tojo-cho, Tennoji-ku, Osaka 543-0026, Japan
Telephone: +81-6-6771-3147
Fax: +81-6-6772-0676
Email: yamamotosei@iph.osaka.jp

Supplementary Figure S1.

Maximum-likelihood phylograms based on nucleotide sequences of a novel human reovirus, the MRV-2 Osaka strain, detected in Osaka City, Japan. ***A*** L1 segment (3,750 bp) with general time reversible (GTR) plus gamma (+G) plus invariable sites (+I) model. ***B*** L2 segment (3,861 bp) with GTR +G +I model. ***C*** L3 segment(3,819 bp) with GTR +G +I model. ***D*** M1 segment (2,202 bp)) with GTR +G +I model. ***E*** M2 segment (1,959 bp) with Tamura 3-parameter +G +I model. ***F*** M3 segment (1,866 bp) with GTR +G +I model. ***G*** S3 segment (834 bp) with GTR +G model. ***H*** S4 segment (1,029 bp) with Tamura 3-parameter +G model. Solid squares indicate the strains detected in this study. Each strain ID consisting of MRV type, accession number, host species, three-letter country name abbreviation, and detection year was based on data which was obtained from GenBank in February 2019. Numbers at the nodes indicate the bootstrap support values, which are given as a percentage of 1,000 replicates (values less than 80% are omitted). Scale bar indicates genetic distances (nucleotide substitutions per site). The GenBank/EMBL/DDBJ accession numbers for the sequences of the MRV-2 Osaka1994, Osaka2005, and Osaka 2014 strains are LC476895–LC476904, LC476905–LC476914, and LC476915–LC476924, respectively.

Supplementary Figure S1 (Yamamoto *et al.*)

A **L1**

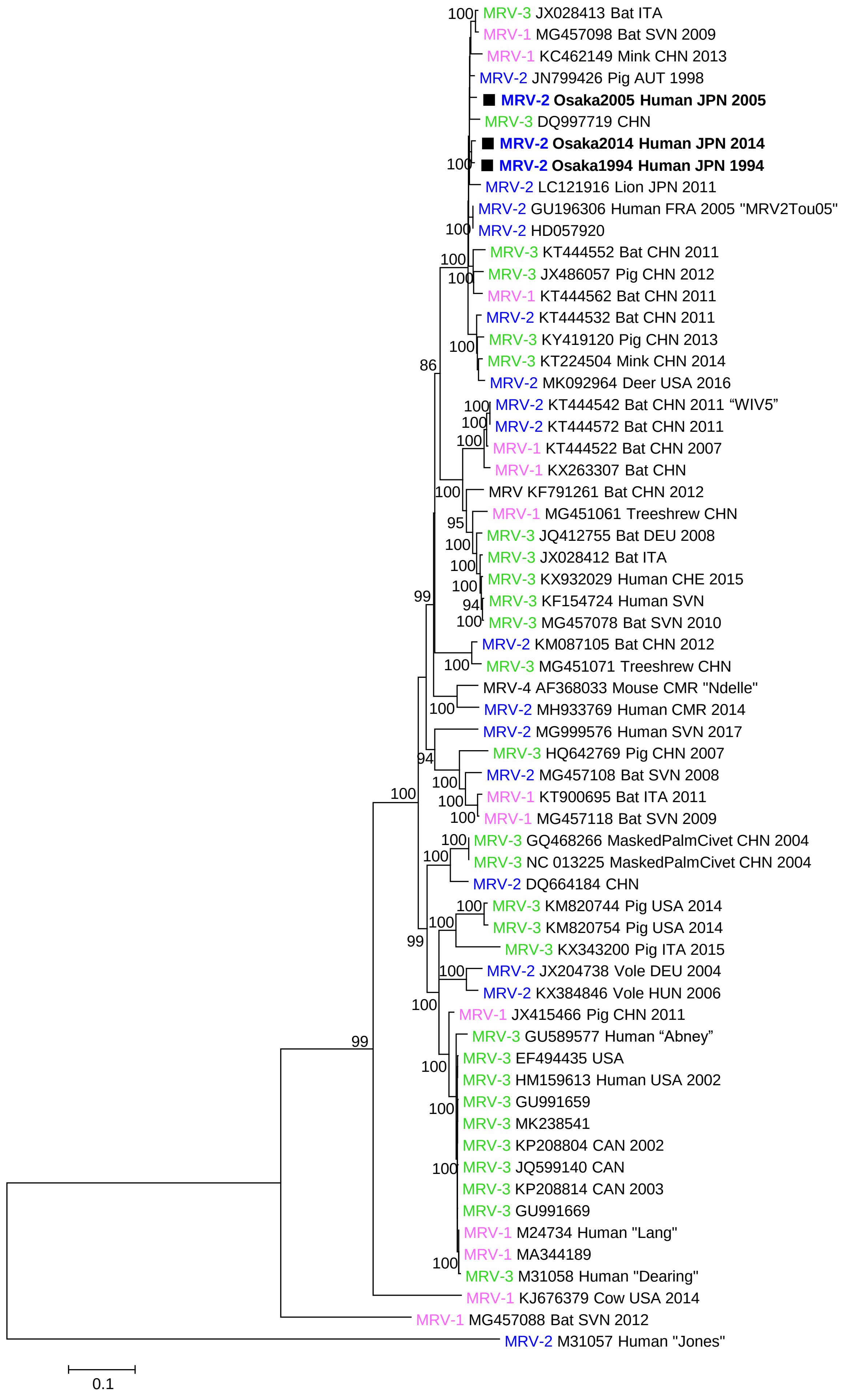

Supplementary Figure S1 (Yamamoto *et al.*)

***B* L2**

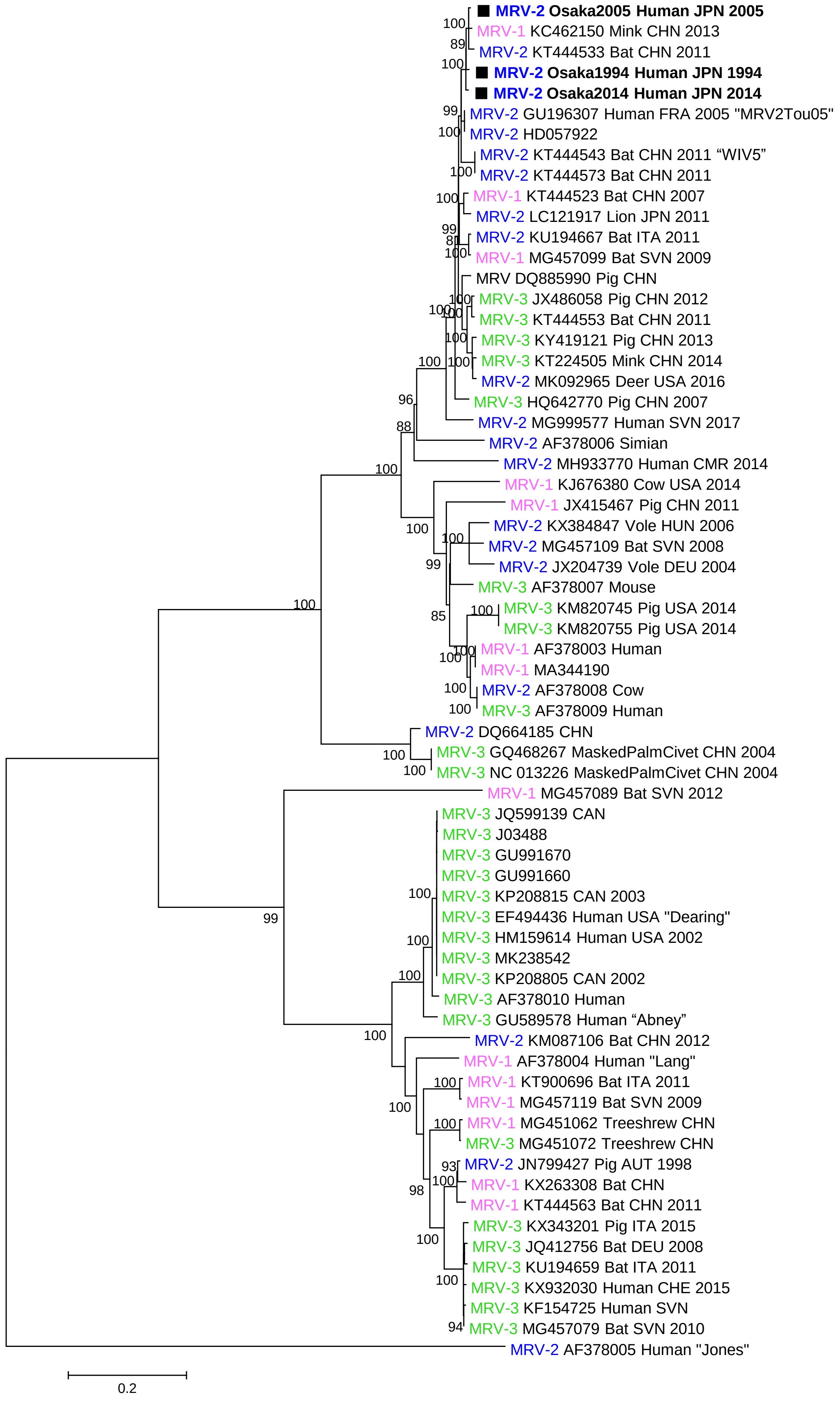

Supplementary Figure S1 (Yamamoto *et al.*)

C L3

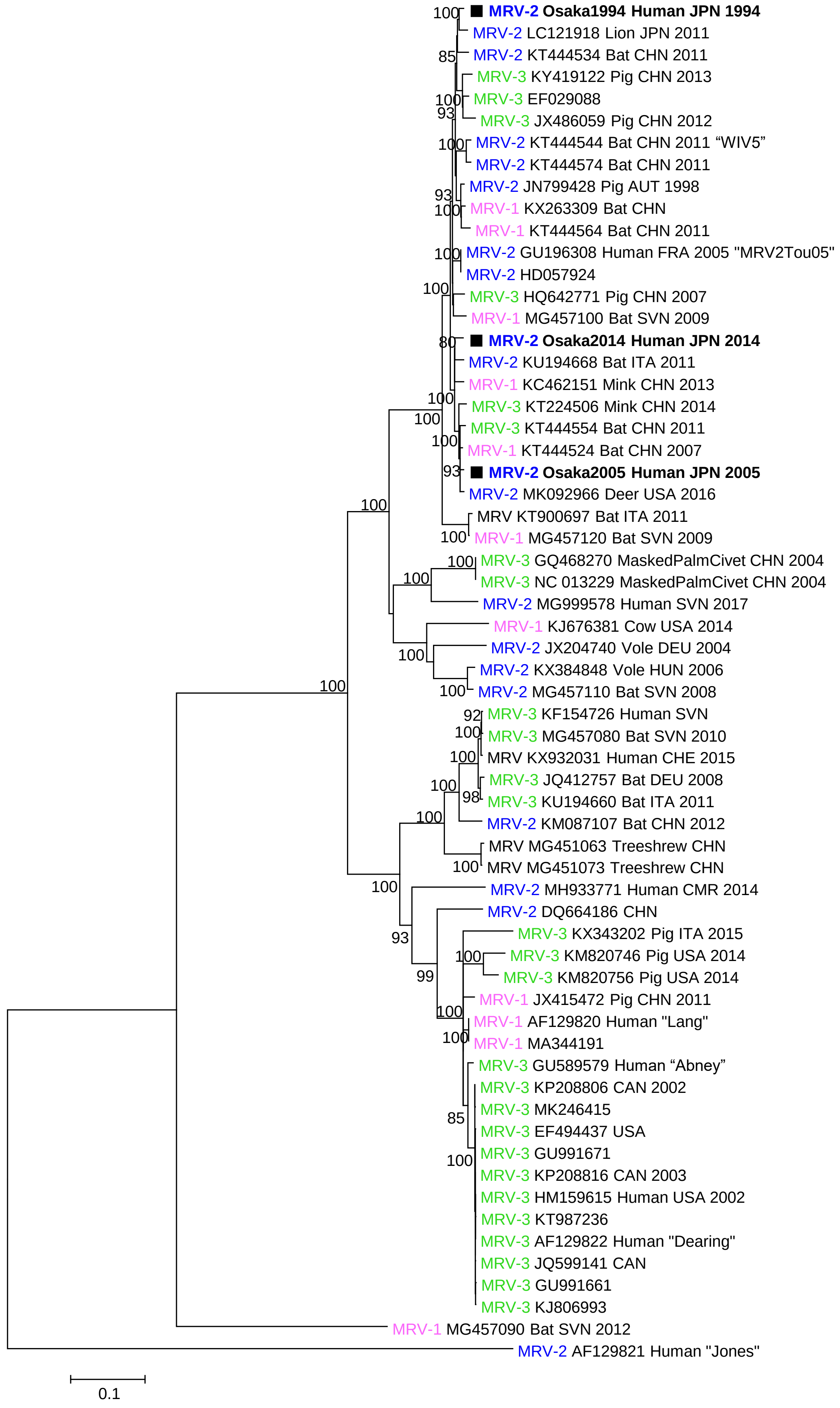

Supplementary Figure S1 (Yamamoto *et al.*)

D M1

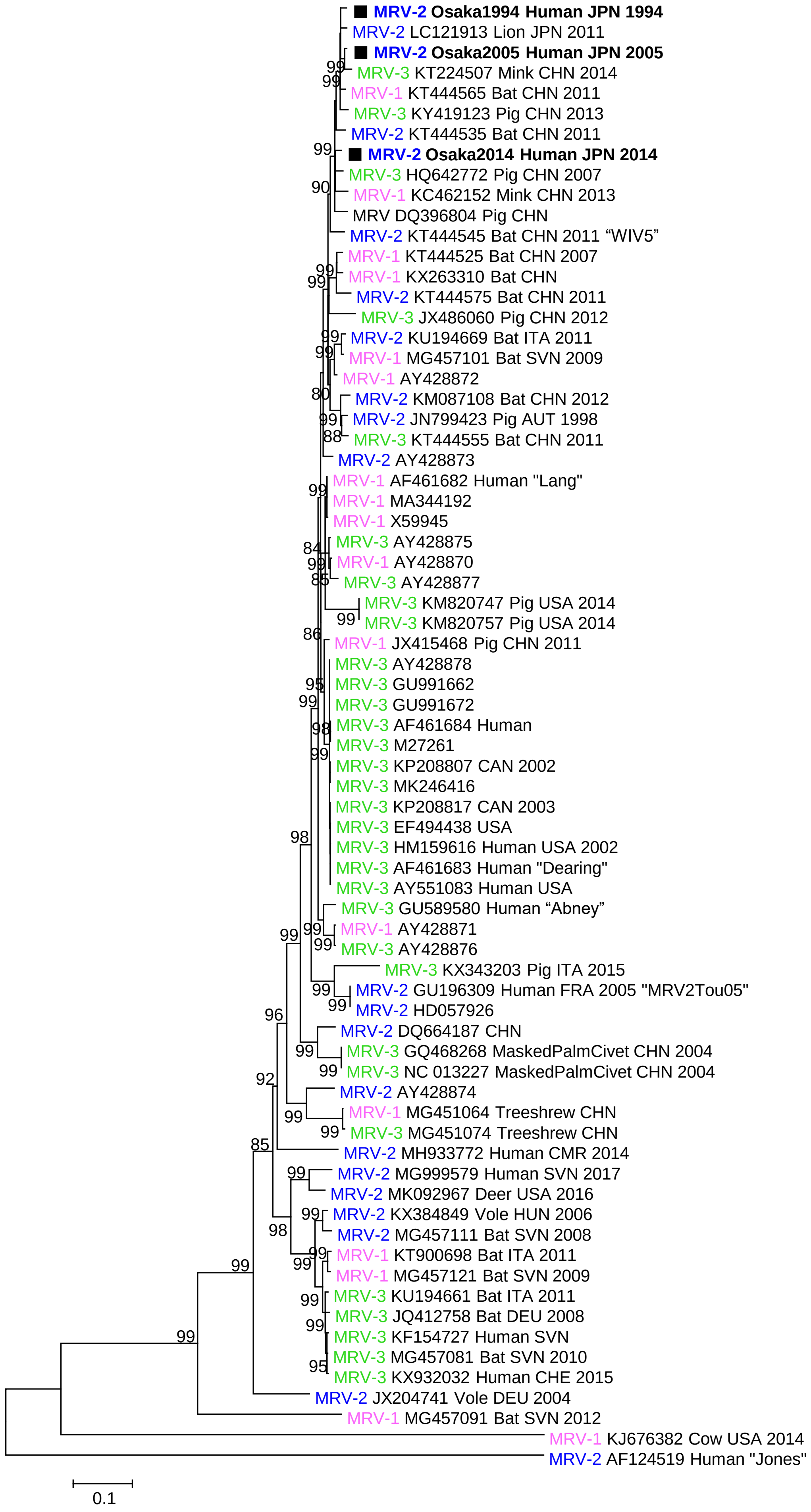

***E* M2**

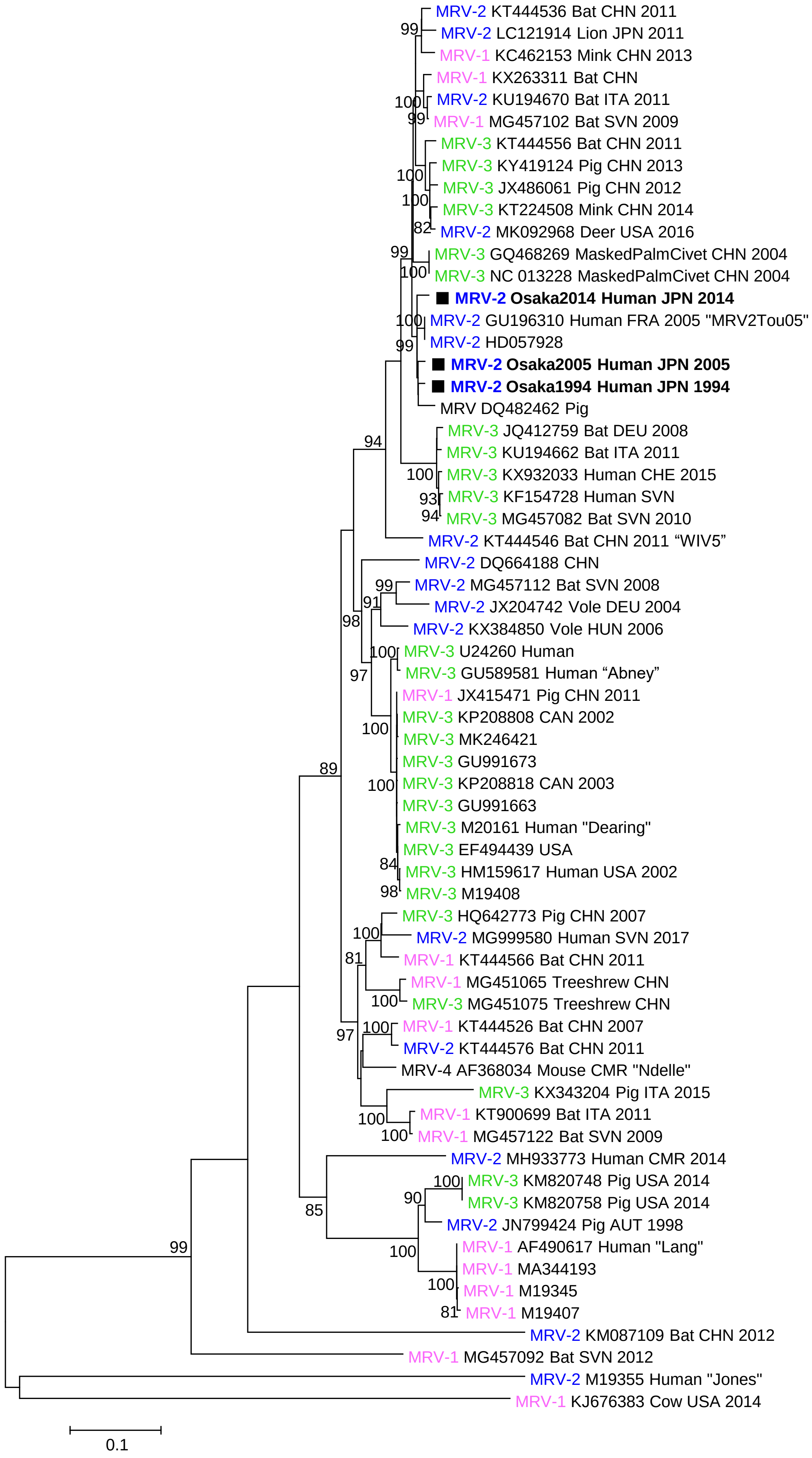
MRV-2 KT444536 Bat CHN 2011
MRV-2 LC121914 Lion JPN 2011
MRV-1 KC462153 Mink CHN 2013
MRV-1 KX263311 Bat CHN
MRV-2 KU194670 Bat ITA 2011
MRV-1 MG457102 Bat SVN 2009
MRV-3 KT444556 Bat CHN 2011
MRV-3 KY419124 Pig CHN 2013
MRV-3 JX486061 Pig CHN 2012
MRV-3 KT224508 Mink CHN 2014
MRV-2 MK092968 Deer USA 2016
MRV-3 GQ468269 MaskedPalmCivet CHN 2004
MRV-3 NC 013228 MaskedPalmCivet CHN 2004
MRV-2 Osaka2014 Human JPN 2014
MRV-2 GU196310 Human FRA 2005 "MRV2Tou05"
MRV-2 HD057928
MRV-2 Osaka2005 Human JPN 2005
MRV-2 Osaka1994 Human JPN 1994
MRV DQ482462 Pig
MRV-3 JQ412759 Bat DEU 2008
MRV-3 KU194662 Bat ITA 2011
MRV-3 KX932033 Human CHE 2015
MRV-3 KF154728 Human SVN
MRV-3 MG457082 Bat SVN 2010
MRV-2 KT444546 Bat CHN 2011 "WIV5"
MRV-2 DQ664188 CHN
MRV-2 MG457112 Bat SVN 2008
MRV-2 JX204742 Vole DEU 2004
MRV-2 KX384850 Vole HUN 2006
MRV-3 U24260 Human
MRV-3 GU589581 Human "Abney"
MRV-1 JX415471 Pig CHN 2011
MRV-3 KP208808 CAN 2002
MRV-3 MK246421
MRV-3 GU991673
MRV-3 KP208818 CAN 2003
MRV-3 GU991663
MRV-3 M20161 Human "Dearing"
MRV-3 EF494439 USA
MRV-3 HM159617 Human USA 2002
MRV-3 M19408
MRV-3 HQ642773 Pig CHN 2007
MRV-2 MG999580 Human SVN 2017
MRV-1 KT444566 Bat CHN 2011
MRV-1 MG451065 Treeshrew CHN
MRV-3 MG451075 Treeshrew CHN
MRV-1 KT444526 Bat CHN 2007
MRV-2 KT444576 Bat CHN 2011
MRV-4 AF368034 Mouse CMR "Ndelle"
MRV-3 KX343204 Pig ITA 2015
MRV-1 KT900699 Bat ITA 2011
MRV-1 MG457122 Bat SVN 2009
MRV-2 MH933773 Human CMR 2014
MRV-3 KM820748 Pig USA 2014
MRV-3 KM820758 Pig USA 2014
MRV-2 JN799424 Pig AUT 1998
MRV-1 AF490617 Human "Lang"
MRV-1 MA344193
MRV-1 M19345
MRV-1 M19407
MRV-2 KM087109 Bat CHN 2012
MRV-1 MG457092 Bat SVN 2012
MRV-2 M19355 Human "Jones"
MRV-1 KJ676383 Cow USA 2014
99
100
99
100
100
82
99
100
100
99
94
100
93
94
98
99
91
97
100
100
100
84
98
89
100
81
100
97
100
100
100
85
100
90
100
100
81
99
0.1

F M3

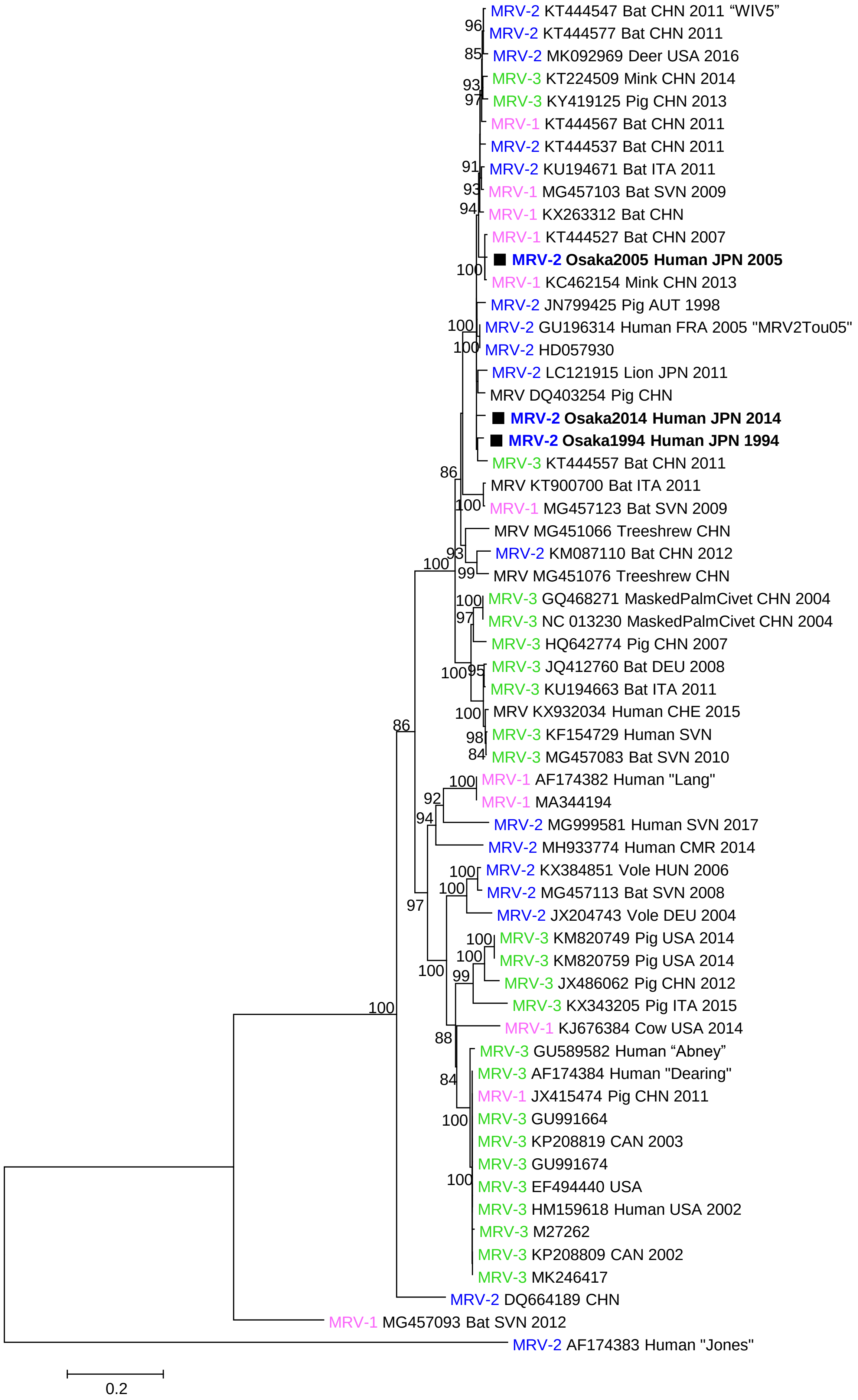
MRV-2 KT444547 Bat CHN 2011 "WIV5"
MRV-2 KT444577 Bat CHN 2011
MRV-2 MK092969 Deer USA 2016
MRV-3 KT224509 Mink CHN 2014
MRV-3 KY419125 Pig CHN 2013
MRV-1 KT444567 Bat CHN 2011
MRV-2 KT444537 Bat CHN 2011
MRV-2 KU194671 Bat ITA 2011
MRV-1 MG457103 Bat SVN 2009
MRV-1 KX263312 Bat CHN
MRV-1 KT444527 Bat CHN 2007
MRV-2 Osaka2005 Human JPN 2005
MRV-1 KC462154 Mink CHN 2013
MRV-2 JN799425 Pig AUT 1998
MRV-2 GU196314 Human FRA 2005 "MRV2Tou05"
MRV-2 HD057930
MRV-2 LC121915 Lion JPN 2011
MRV DQ403254 Pig CHN
MRV-2 Osaka2014 Human JPN 2014
MRV-2 Osaka1994 Human JPN 1994
MRV-3 KT444557 Bat CHN 2011
MRV KT900700 Bat ITA 2011
MRV-1 MG457123 Bat SVN 2009
MRV MG451066 Treeshrew CHN
MRV-2 KM087110 Bat CHN 2012
MRV MG451076 Treeshrew CHN
MRV-3 GQ468271 MaskedPalmCivet CHN 2004
MRV-3 NC 013230 MaskedPalmCivet CHN 2004
MRV-3 HQ642774 Pig CHN 2007
MRV-3 JQ412760 Bat DEU 2008
MRV-3 KU194663 Bat ITA 2011
MRV KX932034 Human CHE 2015
MRV-3 KF154729 Human SVN
MRV-3 MG457083 Bat SVN 2010
MRV-1 AF174382 Human "Lang"
MRV-1 MA344194
MRV-2 MG999581 Human SVN 2017
MRV-2 MH933774 Human CMR 2014
MRV-2 KX384851 Vole HUN 2006
MRV-2 MG457113 Bat SVN 2008
MRV-2 JX204743 Vole DEU 2004
MRV-3 KM820749 Pig USA 2014
MRV-3 KM820759 Pig USA 2014
MRV-3 JX486062 Pig CHN 2012
MRV-3 KX343205 Pig ITA 2015
MRV-1 KJ676384 Cow USA 2014
MRV-3 GU589582 Human "Abney"
MRV-3 AF174384 Human "Dearing"
MRV-1 JX415474 Pig CHN 2011
MRV-3 GU991664
MRV-3 KP208819 CAN 2003
MRV-3 GU991674
MRV-3 EF494440 USA
MRV-3 HM159618 Human USA 2002
MRV-3 M27262
MRV-3 KP208809 CAN 2002
MRV-3 MK246417
MRV-2 DQ664189 CHN
MRV-1 MG457093 Bat SVN 2012
MRV-2 AF174383 Human "Jones"
0.2

Supplementary Figure S1 (Yamamoto *et al.*)

G S3

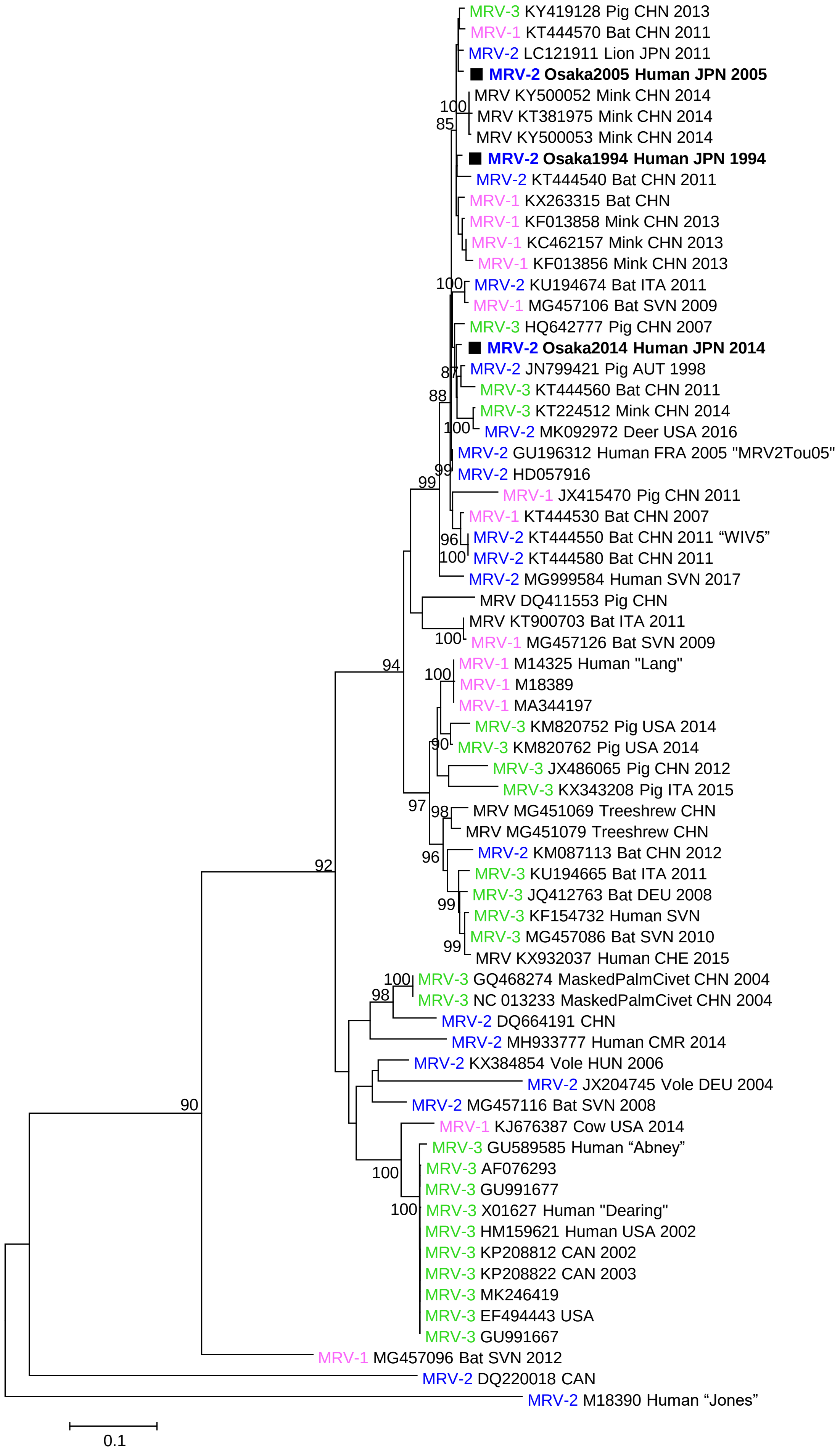

Supplementary Figure S1 (Yamamoto *et al.*)

H S4

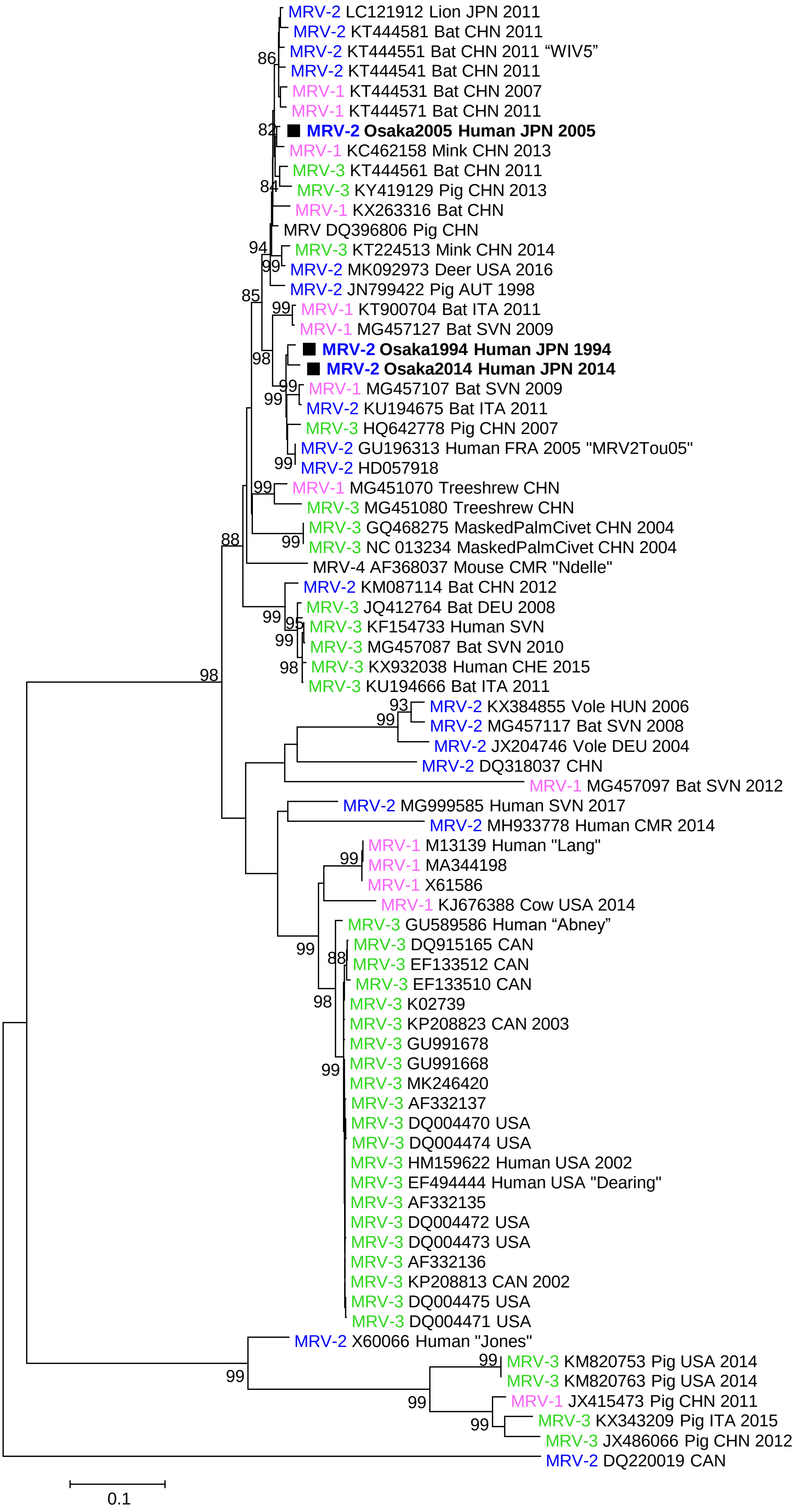

Supplement: Supplementary file 1 — Supplementary information [file 41598_2020_58003_MOESM1_ESM.pdf]
